# Chronic Ethanol Exposures Leads to a Negative Affective State in Female Rats That Is Accompanied by a Paradoxical Decrease in Ventral Hippocampus Excitability

**DOI:** 10.3389/fnins.2021.669075

**Published:** 2021-04-30

**Authors:** Eva C. Bach, James W. Morgan, Sarah E. Ewin, Samuel H. Barth, Kimberly F. Raab-Graham, Jeffrey L. Weiner

**Affiliations:** ^1^Department of Physiology and Pharmacology, Wake Forest School of Medicine, Winston-Salem, NC, United States; ^2^Department of Anesthesia, Wake Forest School of Medicine, Winston-Salem, NC, United States

**Keywords:** alcohol use disorder, chronic intermittent ethanol exposure, negative affective behavior, electrophysiology, hippocampus

## Abstract

Alcohol use disorder (AUD) differentially impacts men and women and a growing body of evidence points to sex-dependent adaptations in a number of brain regions. In a prior study, we explored the effect of a chronic intermittent ethanol exposure (CIE) model of AUD on neuronal and molecular adaptations in the dorsal and ventral domains of the hippocampus (dHC and vHC, respectively) in male rats. We found the vHC to be particularly sensitive to CIE, showing an increase in neuronal excitability and synaptic proteins associated with augmented excitation. These findings were accompanied by a CIE-dependent increase in anxiety-like behaviors. To explore sex-dependent adaptations in the hippocampus, we conducted a similar study in female rats. CIE-treated female rats showed a relatively modest increase in anxiety-like behaviors along with a robust increase in depressive-like measures. Despite both sexes showing clear evidence of a negative affective state following CIE, the vHC of females showed a decrease, rather than an increase, in neuronal excitability. In line with the reduced sensitivity to neural adaptations in the dHC of male rats, we were unable to identify any functional changes in the dHC of females. The functional changes of the vHC in female rats could not be explained by altered expression levels of a number of proteins typically associated with changes in neuronal excitability. Taken together, these findings point to sex as a major factor in CIE-dependent hippocampal adaptations that should be explored further to better understand possible gender differences in the etiology and treatment of AUD.

## Introduction

Alcohol use disorder (AUD) disproportionately impacts men, but this gender gap is closing (Grant et al., 2015; White et al., 2015). Considering the enhanced vulnerability of females to a host of medical consequences of excessive alcohol consumption, including those of the central nervous system, this is an alarming trend (Ammendola et al., 2000; Mann et al., 2005). The underlying causes of these sex differences in the neurobiological symptoms among individuals suffering from AUD remain poorly understood and need to be addressed as they may reveal important differences in how AUD should be treated in men and women.

It has long been appreciated that AUD is highly comorbid with mood disorders including major depressive disorder, anxiety disorders, as well as post-traumatic stress disorder (Grant et al., 2015; Gilpin and Weiner, 2017). Negative affective states associated with these comorbidities facilitate the development and persistence of AUD. One prevailing theory is that the progression from recreational use to compulsive misuse is driven by a shift from experiencing positive reinforcement associated with acute drinking to negative reinforcement during withdrawal. A negative affective state (e.g., elevated levels of stress and anxiety) perpetuates a desire to drink in order to alleviate the aversive symptoms that emerge during periods of abstinence (Becker, 2008; Koob and Le Moal, 2008; Vengeliene et al., 2008). Stress in turn also feeds into, and negatively impacts, relapse behavior in treatment-seeking individuals suffering from AUD (Sinha et al., 2009, 2011). While negative affective comorbidities are common in both sexes, women with AUD have a higher incidence of major depressive disorder, substance-induced depression, and are more prone to drink in response to negative emotional situations (Zilberman et al., 2003; Karpyak et al., 2016, 2019). Differences in regional response patterns to alcohol throughout the brain are thought to play an integral role in these sex-dependent disparities.

One brain region that has received significant attention for its role in AUD is the hippocampus. This interest has, in part, been driven by the well-established role of the hippocampus in cognitive processes (i.e., memory) that are also disrupted in individuals suffering from AUD (Kopera et al., 2012; Le Berre et al., 2017; Zahr and Pfefferbaum, 2017). In rodent models of AUD, hippocampus dependent memory deficits are associated with hippocampal hyperexcitability that is driven by both an increase in excitatory drive and a decrease in inhibitory neurotransmission (Roberto et al., 2002; Nelson et al., 2005). Apart from its cognitive functions, however, the hippocampus is increasingly being appreciated as a brain region integrally involved in modulating affective states which play such a pivotal role in AUD (Fanselow and Dong, 2010; Ciocchi et al., 2015; Yang and Wang, 2017). The role of the hippocampus in AUD and affective disorders has also been supported by clinical studies showing a reduction in hippocampal volume in individuals suffering from these conditions (Chanraud et al., 2009; Zahr and Pfefferbaum, 2017; Pandey et al., 2018). Volume reductions in individuals with affective disorders are predominantly localized to the anterior (analogous to the rodent ventral) hippocampus, a finding in line with studies in rodents that have identified the ventral hippocampus (vHC) as the primary region involved in modulating affective states (Fanselow and Dong, 2010; Malykhin et al., 2010). The vHC is integrally involved in the stress response and this response is bidirectionally modulated by inputs from the basolateral amygdala (BLA), a region well established for its role in addiction and affective behaviors (Sanders and Shekhar, 1995; Lack et al., 2007). The regional difference in hippocampal function and input specificity prompted our prior study comparing neuroadaptations of the dHC and vHC in male rats following withdrawal from chronic intermittent ethanol (CIE) exposure (Ewin et al., 2019). This rodent model of AUD revealed that the ventral, but not dorsal, hippocampus is hyperexcited following alcohol withdrawal and these neurobiological changes were accompanied by an increase in anxiety-like behaviors. Notably, this study and others focused on the effects of chronic alcohol on hippocampal function have largely been limited to male rodents.

The accumulating evidence identifying distinct sex-dependent behavioral and neural adaptations in response to AUD and negative affective states led us expand upon our prior male study to explore these adaptations in female rats during withdrawal from CIE. Despite the robust CIE-dependent anxiogenic phenotypes we previously identified in male rats, measures of anxiety-like behaviors in female rats were less pronounced and only observed on more sensitive measures of anxiety-like behavior. Notably, female CIE-treated rats did exhibit a robust depressive-like phenotype. Our comparison of functional changes across hippocampal regions in female rats affirmed the general finding that the vHC is more sensitive to functional adaptations in excitatory neurotransmission than the dHC in response to CIE treatment. Strikingly, while we previously observed a CIE-dependent increase in excitatory neurotransmission in the vHC of male rats, females showed a decrease in excitatory neurotransmission in this same region. We also explored expression levels of a variety of proteins commonly associated with changes in excitatory or inhibitory neurotransmission, but these did not provide clear insight into the possible mechanisms underlying the observed functional changes of the hippocampus.

## Experimental Procedures

### Animals

Female Long Evans rats were purchased from Envigo, IN and arrived at 175–200 g. Upon arrival, rats were singly housed in clear cages (25.4 cm × 45.7 cm), maintained on a reverse 12 h:12 h light dark cycle (lights on at 9 pm) and provided with *ad libitum* food (Prolab RMH 3000, LabDiet: PMI Nutrition International, St. Louis, MO, United States) and water. Animal care procedures were carried out in accordance with the NIH Guide for the Care and Use of Laboratory Animals and were approved by the Wake Forest University Animal Care and Use Committee. A total of 60 (30 Air and 30 CIE) rats were used to conduct all studies. A total of 22 rats for each group were used from three cohorts to conduct behavioral assays. One cohort of animals (Air = 8 rats; CIE = 8 rats) was tested on both the elevated plus = maze (EPM) and the open field test (OFT). Tissue for biochemical (Western Blot) assessments were taken from a subset (Air = 4 rats; CIE = 4 rats) of animals used for behavioral assays. Two separate cohorts of rats (4 rats per group and cohort for a total of Air = 8 rats; CIE = 8 rats) were used for electrophysiological studies.

### Chronic Intermittent Ethanol Exposure

All animals were housed in their home cages and placed in custom-built Plexiglas chambers (Triad Plastics, Winston-Salem, NC, United States). For CIE-treated rats, ethanol vapor was pumped into the chamber for 12 h a day for 10 consecutive days during the light cycle (9 pm to 9 am) while control animals (Air) were exposed only to room air. Animals were weighed daily and tail blood samples were taken throughout the 10-day CIE procedure at 9 am to monitor blood ethanol concentrations (BECs). Following the 10 days of CIE, animals underwent 24 h of withdrawal (no ethanol vapor) and were then run on behavioral assays or sacrificed for electrophysiological or biochemical studies. Because the FST involved a 2-day exposure, testing for this assay was conducted after 48 h of withdrawal.

### Blood Ethanol Determination

Blood ethanol concentrations were always measured on the first, fifth, and tenth day of CIE exposure at 9 am, although in some animals additional BEC measures were taken to ensure stable BECs throughout the duration of treatment. Blood (10 μL) was collected from each rat *via* a tail snip. BECs were determined using a commercially available alcohol dehydrogenase enzymatic assay kit (Carolina Liquid Chemistries Corporation, Brea, CA, United States). Ethanol concentrations were then determined using a spectrophotometer (Molecular Devices Spectra Max). The target range for BECs was 150–225 mg/dL, with the average BEC of all animals included being 163 ± 5.3 mg/dl.

### Forced Swim Test

Air or CIE-treated rats were individually placed in an inescapable circular pool filled with water (23–25°°C) in which they were unable to touch the bottom of the pool. The luminosity at the top of the cylinder was 25 Lux. The test consisted of two sessions, separated by 24 h. The first session (habituation) was 15 min and the second session (test day) was 5 min. Following the assay, rats were dried off using a warmed towel and returned to their home cages. An Ethovision system was used to record the behavior of rats throughout the duration of test session. Analysis for immobility (duration, bouts), distance, and velocity traveled were analyzed offline using Ethovision analysis software.

### Elevated Plus-Maze

To measure unconditioned anxiety-like behavior, behavioral responses of air (control) and CIE-treated rats were tested on a standard elevated plus-maze (Med Associates, St. Albans, VT, United States). The elevated plus-maze was raised 72.4 cm from floor level, with runways measuring 10.2 cm wide by 50.8 cm long. Open runways had 1.3 cm high lips and closed runways were detected *via* infrared sensors attached to the opening of each arm of the maze. Data were obtained and recorded *via* computer interfaced with control units and MED-PC programming (Med Associates). Animals were placed at the junction of the four arms and allowed to freely explore the maze for 5 min. Open arm time and open arm entries were used as measures of anxiety-like behavior and the number of closed arm entries was measured to assess general locomotor activity.

### Open-Field Test

A separate group of air control and CIE-treated rats, general locomotion in a novel environment was measured using an OFT conducted in a Plexiglass chamber (41.5 cm × 41.5 cm × 30 cm). At the start of the test, animals were placed in the center of the chambers equipped with Omnitech Superflex Sensors (Omnitech Electronics, Inc.), which utilize arrays of infrared photodetectors located at regular intervals along each side of the chambers. Exploratory activity in this environment was measured for 30 min, and data were analyzed in 5-min time bins.

### Successive Alleys Test

The successive alleys test consists of a linear series of four alleys designed to be increasingly anxiogenic. The first alley (or zone) is similar to an enclosed arm of the plus-maze, while the three successive alleys (or zones) resemble the open arms of the plus-maze but each becomes narrower, has lower side wall height, and a brighter floor color (Rudebeck et al., 2007; Deacon, 2013; Lahmann et al., 2014). The test is raised 61 cm from the floor level with 36.1-cm walls in zone 1 and 3.81 cm walls in zones 2–4 with a total alley length of 172.72 cm. Each zone is 43.18 cm in length. Zones 1 and 2 are 15.24 cm wide while zone 3 is 10.16 cm and zone 4 is 5.08 cm wide. EthoVision (Noldus) was used to analyze the total distance traveled, velocity, time spent in each zone, the latency to reach zone 4, and head dips in each zone. The assay lasted for a total of 5 min regardless of whether the rat reached zone 4 (most anxiogenic zone). One rat did not reach zone 4 during the 5 min test. Consequently, for our latency analysis we assigned the total duration of the test (300 s) to rats that did not each zone 4 and this occurred in one of our rats.

### Electrophysiology

Electrophysiological experiments were conducted following 24 h of withdrawal from CIE. Animals were deeply anesthetized using isoflurane. Following decapitation, the brain was removed rapidly and suspended in ice-cold cutting artificial cerebral spinal fluid (aCSF) consisting of 85 mM NaCl, 1.25 mM NaH_2_PO_4_, 25 mM NaHCO_3_, 10 mM D-Glucose, 75 mM sucrose, 3 mM KCl, 7 mM MgCl_2_, 0.5 mM CaCl_2_, and 0.6 mM ascorbate bubbled with 95% O_2_ and 5% CO_2_. Transverse slices containing the dHC and vHC were cut at a thickness of 375 μm using a VT1000S Vibratome (Leica Microsystems). Slices were immediately transferred to a holder chamber containing recording aCSF consisting of 125 mM NaCl, 1.25 mM NaH_2_PO_4_, 25 mM NaHCO_3_, 10 mM D-Glucose, 2.5 mM KCl, 1 mM MgCl_2_, and 2 mM CaCl_2_ bubbled with 95% O_2_ and 5% CO_2_. Slices were incubated in the holding chamber at room temperature (21–23°C) for at least 1 h and were subsequently transferred to a recording chamber where they were perfused with oxygenated, heated (32°C) recording aCSF. Borosilicate glass pipettes (0.86 μm), were pulled using a horizontal pipette puller (P-97; Sutter Instrument) to prepare recording electrodes (1–3 MΩ resistance). Recording electrodes were filled with 0.9% saline and placed into the CA1 dendritic layer of the dHC and vHC. A nickel dichromate bipolar stimulating electrode was placed in the Schaffer collateral to pathway to record stimulated extracellular field recordings in the Schaffer collateral-CA1 pathway. For input-output curves, field excitatory post synaptic potentials (fEPSP) were evoked every 10 s at 10, 20, 50, 100, 150, 200, 300, 500, and 700 μA five times per stimulus intensity. All recordings were acquired using an Axoclamp 2B amplifier, digitized (Digidata 1321A; Molecular Devices) and analyzed with pClamp 10.4 software (Molecular Devices).

### Western Blots

Western blot analyses were performed on synaptoneurosomes (SNs) that were obtained from dorsal (dHC) and ventral (vHC) hippocampal slices. Slices were prepared as described in the electrophysiology methods section. dHC and vHC slices were homogenized in buffer [50 mM Tris, pH 7.35; protease and phosphatase inhbitors (Halt, ThermoFisher)] and subsequently filtered through 100 and 5 μm filters to produce SNs (Quinlan et al., 1999; Workman et al., 2013; Niere et al., 2016; Ewin et al., 2019). SNs were centrifuged (14,000 *g*, 20 min, 4°C) to obtain a pellet that was solubilized in RIPA buffer (150 mM NaCl; 10 mM Tris, pH 7.4; 0.1% SDS; 1% Triton X-100; 1% deoxychoate 5 mM EDTA; Halt). The insoluble fraction of SNs was removed by centrifugation at 14,000 *g*, 20 min, 4°C. The soluble fraction was used for immunoblot analysis. 50 μg of protein/sample was separated on a 10% SDS-polyacrylamide gel. The following antibodies were used to visualize the proteins of interest: mouse anti-GluA1 (1:1,000; Neuromab; 75-327); mouse anti-GluA2 (1:1,000; Neuromab; 75-002); rabbit anti-SK2 (1:1,000; Alomone Lab, APC-028); rabbit anti-GluN1 (1:1,000; Alomone Labs; AGC-001); rabbit anti-GluN2B (1:500; Alomone Labs, AGC-003); rabbit anti-GABAAR α1 subunit (1:1,000; Novus, NB300-191) mouse anti-Gephyrin (1:1,000, Synaptic Systems, 147-011). To visualize the proteins, membranes were incubated in fluorescence-conjugated secondary antibodies (AF680; AF800; 1:4,000; LiCor, Lincoln, NE, United States) and imaged using the Odyssey CLx infrared imaging system. For densitometry analysis of proteins, ImageJ software (National Institutes of Health) was used. Protein of interests were normalized to the house keeping gene (Actin) as well as the expression level of the internal control group (AIR-treated rats).

### Statistical Analysis

All statistical analyses, except the ANCOVA analysis, were performed in Sigma Plot 14.0. ANCOVA analysis were conducted using MATLAB R2017a. Behavioral and Western blot data comparing a single factor between treatment groups (Air vs. CIE) were analyzed using unpaired *t*-tests, or Mann–Whitney Rank Sum Tests in the event of non-normally distributed data. The normality of data was assessed using a Shapiro–Wilk test. Zone data on the Successive alleys test were analyzed using a two-way ANOVAs. Electrophysiological data were analyzed using two-way repeated measures ANOVAs. Where noted, *post hoc* analysis was conducted using Bonferroni’s multiple comparison test. A generalized linear model was run to examine the relationship between CIE, fiber volley amplitude and fEPSP slope using an ANCOVA. This analysis was conducted up to a maximum independent variable (fV) for which measures of this covariate could be obtained within 5% in both treatment conditions (Air and CIE). The minimal level of significance was set as *p* < 0.05 for all analyses.

## Results

### Female Rats Display an Increased Negative Affective State During Withdrawal From CIE

**FIGURE 1:**
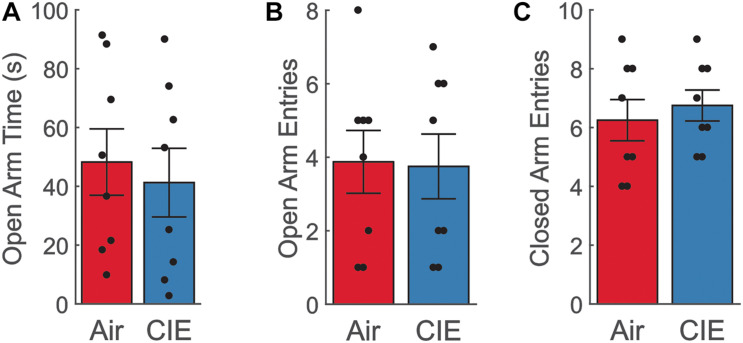
Female CIE-treated rats do not show anxiety-like behavior on the EPM. **(A)** Air (*n* = 8) and CIE-treated (*n* = 8) rats spend similar amount of time in the open arms, **(B)** make similar number of open arm entries, and **(C)** closed arm entries. All data is expressed as the mean ± SEM.

To characterize CIE-dependent anxiety-like behavior in female rats, we first tested our animals on three distinct assays of anxiety-like behavior. Our prior study in male Long Evans rats established a robust anxiogenic phenotype on the EPM at 24-h of withdrawal from CIE (Ewin et al., 2019). When female rats were tested at this same withdrawal timepoint, we saw no evidence of an anxiogenic phenotype on the EPM. Air- and CIE-treated animals spent a similar amount of time in the open arms [Figure 1A; *t*(14) = −0.431; *p* = 0.673], and there was no significant difference between the number of open arm entries [Figure 1B; *t*(14) = −0.102; *p* = 0.920]. To validate that these measures were not biased by differences in locomotor activity, we also compared differences between closed arm entries, a commonly used measure to assess non-specific locomotor activity. No differences in this measure were observed between Air- and CIE-treated animals [Figure 1C; *t*(14) = −0.571; *p* = 0.577].

**FIGURE 2:**
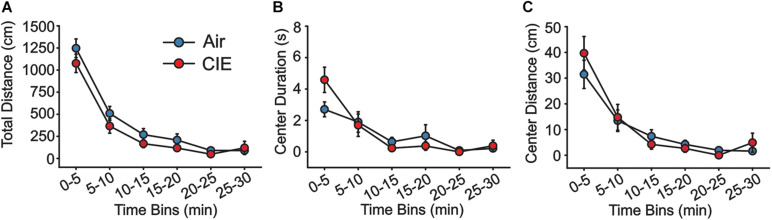
There is no evidence of CIE-induced anxiety-like behavior on the OFT. Air (*n* = 12) and CIE-treated rats (*n* = 12) showed no differences in **(A)** the total distance traveled, **(B)** the amount of time spent, or **(C)** in the distance traveled in the center of the arena. All data are expressed as the mean ± SEM.

As an additional test of anxiety-like behavior, we also explored CIE-dependent behavioral effects on the OFT over the course of 30 min. Using 5 min bins in the analysis, we saw no CIE-dependent main effect on total distance traveled (Figure 2A; *F*_1,22_ = 2.496; *p* = 0.128), although distance traveled decreased significantly in both groups as a function of time (*F*_5,110_ = 87.356; *p* = 0.001). Nonetheless, we found no group interaction between these factors (*F*_5,110_ = 0.668; *p* = 0.649). Both Air and CIE rats spent a similar amount of time in the center (Figure 2B; *F*_1,22_ = 0.112; *p* = 0.741), but spent successively less time in the center throughout the duration of the assay (*F*_5,110_ = 19.965; *p* = 0.001). Again, there was no interaction between treatment group and time for this measure (*F*_5,110_ = 2.201; *p* = 0.059). Finally, we compared the total distance traveled in the center, but found no main effect of group (Figure 2C; *F*_1,22_ = 0.180; *p* = 0.675). While we did observe an effect time (*F*_5,110_ = 30.778; *p* = 0.001), we saw no interaction between time and animal groups (*F*_5,110_ = 0.807; *p* = 0.547).

**FIGURE 3:**
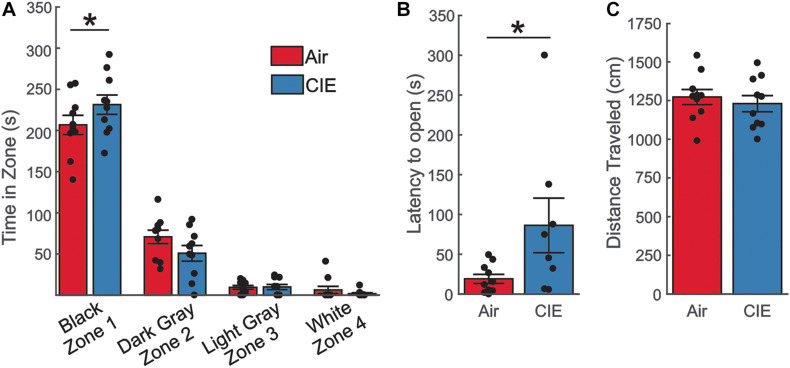
The SAT revealed an anxiety-like phenotype in CIE-treated female rats. **(A)** CIE-treated female rats (*n* = 10) spent more time in the least anxiogenic zone (Black) than Air control rats (*n* = 10), but the two groups spent comparable amounts of time in all other zones. **(B)** CIE-treated rats had a longer latency to venture into the most anxiogenic open zone (White). **(C)** The total distance traveled was similar between Air and CIE-treated female rats. All data are expressed as the mean ± SEM, *p < 0.05.

As a final test of anxiety-like behavior, we employed the SAT. The progressively increasing anxiogenic zones (see methods) of this assay may provide a more sensitive measure to detect subtle differences in anxiety-like behavior and our prior CIE study in male rats identified an anxiogenic phenotype on this assay. We explored measures of anxiety-like behaviors on the SAT in female CIE- and AIR-exposed rats. We first compared the total duration these animals spent in each of the zones. This analysis revealed a main effect of the amount of time the animals spent in each of the four zones, with the animals spending the most time in the enclosed zone (Figure 3A; *F*_3,72_ = 346.1; *p* < 0.0001). Although we found no effect of CIE (*F*_1,72_ = 0.0007061; *p* = 0.9789), we found an interaction between treatment group (CIE vs. Air) and the amount of time spent in each of the zones (*F*_3,72_ = 2.909; *p* = 0.0403). A *post hoc* analysis revealed that CIE-treated rats spent more time in the least anxiogenic zone (Black) [*t*(72) = 2.271; *p* = 0.0261]. When we compared the latency between our animal groups to enter the most anxiogenic zone (Zone 4), we saw a significant difference between Air- and CIE-treated rats (Figure 3B; *U* = 20; *p* = 0.0232; Mann–Whitney-U-test). This effect could not be explained by the animals state of mobility given that there was no significant treatment group difference in total distance traveled [Figure 3C; *t*(18) = 0.600; *p* = 0.556]. Overall, although we did not observe a clear anxiety-like phenotype on the EPM or OFT, the greater amount of time CIE-treated rats chose to spend in the least anxiogenic zone, and the greater latency of these animals to enter the most anxiogenic zone, indicate that female rats do exhibit increases in at least some anxiety-like behaviors during withdrawal from CIE.

**FIGURE 4:**
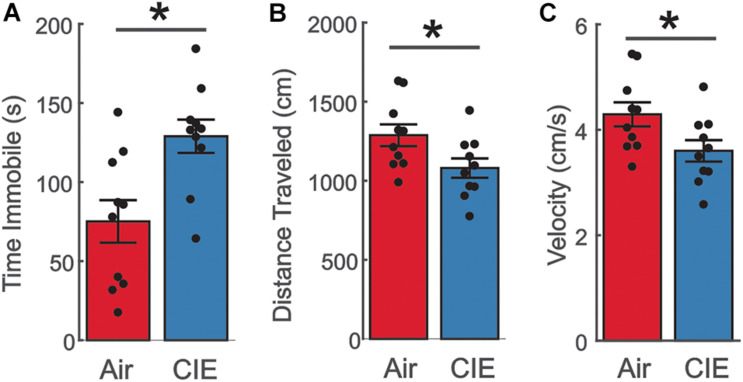
Female CIE-treated rats show a depressive-like phenotype on the FST. **(A)** CIE-treated female rats (*n* = 10) spent more time immobile, **(B)** traveled a shorter distance, and **(C)** had a slower moving velocity than their Air control (*n* = 10) counterparts. All data are expressed as the mean ± SEM *p < 0.05.

The greater prevalence of major depressive disorder in women with AUD (Karpyak et al., 2016, 2019) led us to examine the effect of CIE-treatment on the FST, a behavioral assay particularly sensitive to depressive-like behaviors. CIE-treated female rats spent a greater amount of time immobile [Figure 4A; *t*(18) = −3.146; *p* = 0.00559]. This increase in immobility was accompanied in a reduction in the total distance traveled [Figure 4B; *t*(18) = 2.261; *p* = 0.0364] and overall velocity [Figure 4C; *t*(18) = 2.261; *p* = 0.0364] in female CIE-treated rats. Taken together these findings indicate that CIE-treatment elicits a depressive-like phenotype in female rats.

### CIE Resulted in a Reduction in Neuronal Excitability in the vHC, but Not dHC, of Female Rats

**FIGURE 5:**
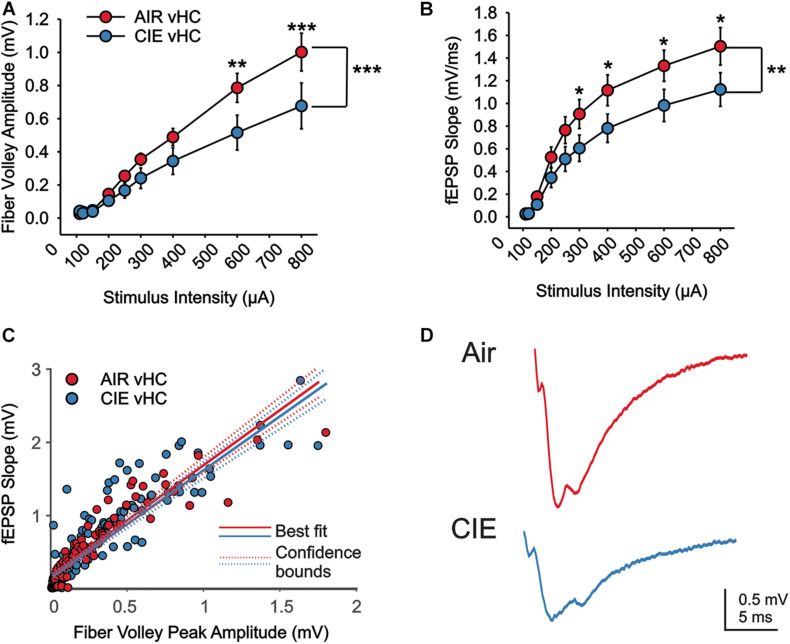
There is a reduction in excitatory neurotransmission in the vHC of CIE-treated rats. There is a CIE-dependent reduction in the stimulus dependent **(A)** fV amplitude and **(B)** fEPSP slope. **(C)** The relative balance between vHC fV amplitude and fEPSP was not impacted by CIE-treatment. **(D)** Representative example traces of fEPSPs acquired from slices of an Air (upper trace) and CIE-treated (lower trace) rat. All data are expressed as the mean ± SEM, *p < 0.05, **p < 0.01 and ***p < 0.001.

We next used extracellular recording methods to examine the effects of CIE on synaptic excitability in the dorsal (dHC) and ventral (vHC) subregions of the hippocampus in female rats. We recorded extracellular field excitatory postsynaptic potentials (fEPSPs) in the Schaffer-collateral-CA1 region of the dHC and vHC. We measured fiber volley (fV) amplitudes and rising slopes of fEPSPs to compare input strength in slices from Air and CIE treated rats (AIR *n* = 13 slices, seven rats; CIE *n* = 11 slices, eight rats) across a range stimulus of intensities in each hippocampal region. In the vHC, we identified no main effect of CIE treatment condition on fiber volley amplitude (Figures 5A,D; *F*_1,16_ = 3.935; *p* = 0.065; Two Way Repeated Measures ANOVA) with a main effect on stimulus intensity (*F*_1,8_ = 75.761; *p* < 0.001; Two Way Repeated Measures ANOVA). We further found an interaction between CIE treatment condition and stimulus intensity (*F*_1,8_ = 4.051; *p* < 0.0001). Our *post hoc* analysis revealed a CIE-dependent decrease in fiber volley amplitude at the two highest stimulus intensities (*p* = 0.001 for 500 μA; *p* < 0.001 for 700 μA). When we compared the fEPSP slope across stimulus intensities, we identified no main effect of CIE treatment (Figures 5B,D; *F*_1,22_ = 2.893; *p* = 0.1031; Two Way Repeated Measures ANOVA), but a significant effect of stimulus intensity (*F*_8,176_ = 110.1; *p* < 0.0001; Two Way Repeated Measures ANOVA). We also found an interaction between stimulus intensity and CIE treatment (*F*_8,176_ = 2.763; *p* < 0.0067; Two Way Repeated Measures ANOVA). A *post hoc* analysis revealed that fEPSP slopes were significantly lower for the four highest stimulus intensities (*p* = 0.0448 for 200 μA; *p* = 0.0258 for 300 μA; *p* = 0.0203 for 500 μA; *p* = 0.0116 for 700 μA). To further explore how CIE treatment impacts fEPSP slope as a function of fV amplitude, we used a linear regression model to conduct an analysis of covariance (ANCOVA). Using this analysis we did not identify a CIE-dependent difference in the relationship between fV amplitude and fEPSP slope (Figures 5C,D; *F*_1,212_ = 0.1; *p* = 0.749). Taken together the decrease in fV amplitude (associated with presynaptic changes) and fEPSP slope (associated with postsynaptic changes) in CIE-treated rats indicates a decrease in excitatory neurotransmission, but that the relationship between pre- and postsynaptic excitability remains unaffected by CIE.

**FIGURE 6:**
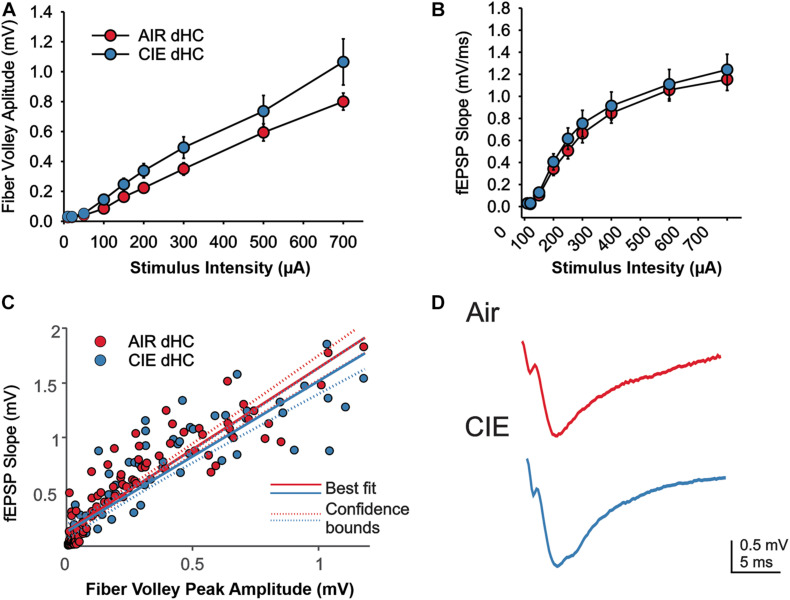
Excitatory neurotransmission in the dHC was not impacted by CIE treatment. We found no effect of CIE on the stimulus-dependent **(A)** fV amplitude and **(B)** fEPSP slope, or **(C)** the relative balance between dHC fV amplitude and fEPSP. **(D)** Representative example traces of fEPSPs acquired from slices of an Air (upper trace) and CIE-treated (lower trace) rat. All data are expressed as the mean ± SEM.

We also compared extracellular field responses between Air- and CIE-treated animals in the dHC. In the dHC we found no main effect of CIE treatment on fV amplitude (Figures 6A,D; *F*_1,18_ = 2.944; *p* = 0.1034; Two Way Repeated Measures ANOVA), but a main effect on stimulus intensity (*F*_1,8_ = 75.761; *p* < 0.001; Two Way Repeated Measures ANOVA). We found a trend toward a significant interaction, between stimulus intensity and CIE treatment (*F*_8,144_ = 1.967; *p* = 0.0547; Two Way Repeated Measures ANOVA). Our comparison between treatment groups on fEPSP slopes in the dHC yielded no main treatment effect (Figures 6B,D; *F*_1,18_ = 0.3521; *p* = 0.5603; Two Way Repeated Measures ANOVA), but an effect of stimulus intensity (*F*_8,144_ = 130.7; *p* < 0.0001; Two Way Repeated Measures ANOVA). We further saw no interaction between treatment group and stimulus intensity (*F*_8,144_ = 0.238; *p* = 0.983; Two Way Repeated Measures ANOVA). We again, used an ANCOVA to gain a better understanding of the relationship between fV amplitude and fEPSP slope. We did not see an effect of CIE on this relationship (Figures 6C,D; *F*_1,156_ = 2.14; *p* = 0.1456). Taken together these findings indicate that the CIE treatment has minimal effects on excitatory neurotransmission in the dHC of female rats.

### CIE Had no Effect on the Expression of Synaptic Proteins That Influence Excitatory Neurotransmission, but GluA1 Subunit Expression Was Increased in the dHC Following CIE

**FIGURE 7:**
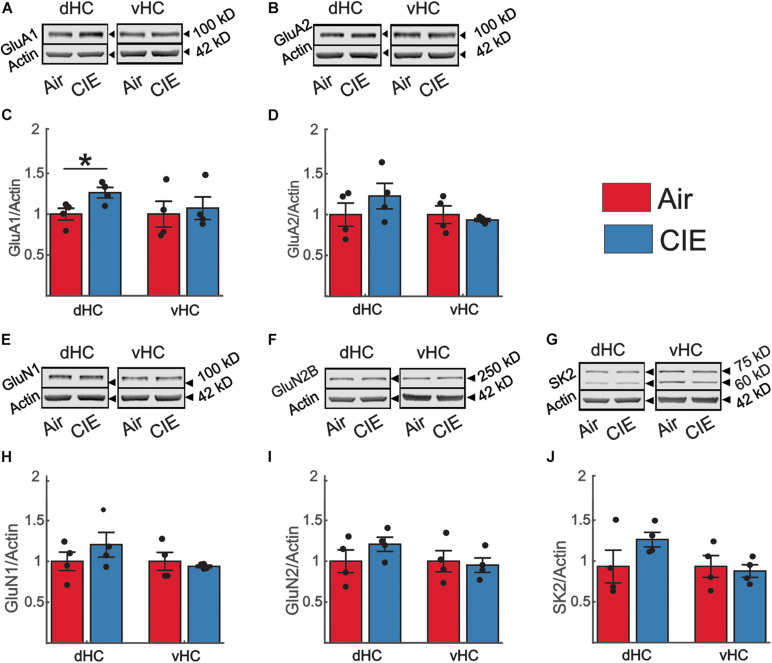
CIE does not impact the expression levels of proteins commonly associated with alterations in excitatory neurotransmission in the vHC. However, in the dHC, the GluA1 subunit is upregulated. Representative western blot examples illustrating the expression of **(A)** the GluA1 and **(B)** GluA2 subunit of the AMPA receptor, **(E)** the GluN1, **(F)** and GluN2 subunit of the NMDA receptor and **(G)** SK2. In panels **(A,B,E–G)** the corresponding expression of actin (normalization protein) is shown in the lower blot panel of each protein of interest. **(C,D,H–J)** Group data, normalized to actin and the internal control group (Air), for the **(C)** GluA1 (Air *n* = 4, CIE *n* = 4), **(D)** GluA2 (Air *n* = 4, CIE *n* = 4), **(H)** GluN1 (Air *n* = 4, CIE *n* = 4), **(I)** GluN2B subunit (Air *n* = 4, CIE *n* = 4) and **(J)** the SK2 channel (Air *n* = 4, CIE *n* = 4) illustrating an increase in the expression of the GluA1 subunit (Air *n* = 4, CIE *n* = 4) in the dHC without changes in other proteins in the dHC and vHC of Air and CIE-treated rats. All data are expressed as the mean ± SEM, *p < 0.05.

To begin to address possible mechanisms responsible for the changes in vHC excitatory neurotransmission following CIE treatment in female rats, we evaluated the expression level of several proteins known to influence hippocampal neuronal excitability by examining synaptoneurosomal (SN) fractions prepared from the dHC and vHC. We first examined two AMPA receptor subunits that are highly expressed in the hippocampus, GluA1 and GluA2 (Kohama and Urbanski, 1997). In female CIE-treated rats, we found no significant change in the expression level of the GluA1 subunit in the vHC [*t*(6) = 0.35; *p* = 0.738; Figures 7A,C], but we did detect an increase in the expression of this subunit in the dHC [*t*(6) = 2.71; *p* = 0.035; Figures 7A,C]. In either the vHC [*t*(6) = 0.60; *p* = 0.570; Figures 7B,D] or the dHC [*t*(6) = 1.08; *p* = 0.161; Figures 7B,D], we did not identify a change in the expression level of the GluA2 subunit. We next assessed the expression level of the obligatory NMDA receptor subunit GluN1, typically viewed as proxy of the number of NMDA receptors (Paoletti and Neyton, 2007). We found no differences in the expression level of GluN1 subunits in the vHC [*t*(6) = 0.56; *p* = 0.597; Figures 7E,H] or dHC [*t*(6) = 1.08; *p* = 0.321; Figures 7E,H] between Air and CIE-treated rats. Subunit-specific changes can also drive functional changes, and the GluN2B subunit is a particularly prevalent subunit associated with excitatory neuroplasticity. Again, we found no difference between treatment groups of the GluN2B subunit expression level in the vHC [*t*(6) = 0.31; *p* = 0.770; Figures 7F,I] or dHC [*t*(6) = 1.26; *p* = 0.253; Figures 7F,I]. The small conductance calcium-activated potassium channel (SK2) modulates neuronal excitability and our prior study exploring the role of CIE in male rats revealed a decrease in the expression of SK2 only in the vHC (Ewin et al., 2019), consistent with the observed increase in excitatory neurotransmission in this region. This led us to explore CIE-dependent alteration in SK2 expression in female rats. However, we found no difference between our Air and CIE-treated rats in the expression of SK2 channel in the dHC [*t*(6) = 1.50; *p* = 0.183; Figures 7G,J] or vHC [*t*(6) = 0.36; *p* = 0.730; Figures 7G,J].

**FIGURE 8:**
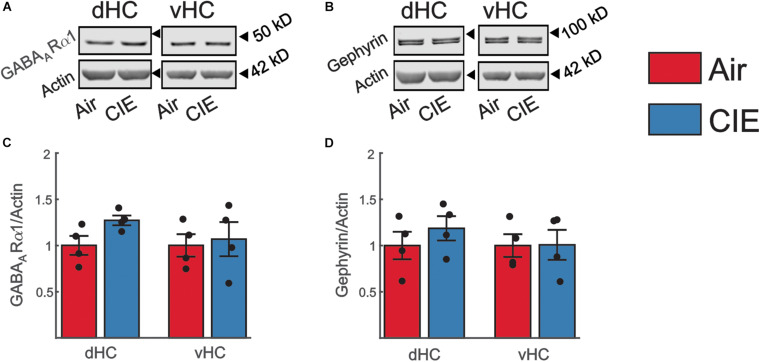
Expression levels of proteins commonly associate with changes in inhibitory neurotransmission are not impacted by CIE-treatment in the dHC or vHC. Representative western blot examples illustrating the expression of **(A)** the GABA_*A*_Rα1 subunit or **(B)** Gephyrin. The corresponding expression of actin (normalization protein) is shown in the lower blot panel of each protein of interest. **(C,D)** Group data, normalized to actin and the internal control group (Air), for **(C)** the GABA_*A*_Rα1 subunit (Air *n* = 4, CIE *n* = 4) and for **(D)** Gephyrin (Air *n* = 4, CIE *n* = 4) illustrating no change in the expression of these proteins in the dHC and vHC following CIE-treatment. All data are expressed as the mean ± SEM.

To address the possibility that our observed decrease in excitatory neurotransmission in the vHC was the result of an increase in inhibitory neurotransmission, we also examined the effect of CIE on two prominent proteins that regulate GABAergic inhibitory neurotransmission. In the present study we focused our attention on the α subunit of the GABA_*A*_R and the GABA_*A*_ anchoring protein gephyrin due to their prominent role in modulating neuronal excitability in animal models of alcohol/substance use disorders (Liang and Olsen, 2014; Barker and Hines, 2020). We identified no significant changes in the expression of the α subunit of the GABA_*A*_R in the vHC [*t*(6) = 0.30; *p* = 0.772; Figures 8A,C] or dHC [*t*(6) = 2.35; *p* = 0.057; Figures 8A,C]. The expression level of gephyrin, similarly remained unaffected by CIE-treatment in the vHC [*t*(6) = 0.04; *p* = 0.971; Figures 8B,D] or dHC [*t*(6) = 0.94; *p* = 0.385; Figures 8B,D].

## Discussion

We recently examined the effects of CIE, a well-validated rodent model of AUD, on anxiety-like behaviors and hippocampal excitability in male Long Evans rats. We discovered that, at a withdrawal timepoint associated with significant increases in anxiety measures, hippocampal synaptic excitability was increased in the ventral, but not dorsal, domain of this brain region and these changes were accompanied by alterations in the expression of GluA2 and small-conductance calcium-activated potassium (SK) channel subunits. Here, we used the same CIE procedure to examine behavioral and hippocampal adaptations in female rats, which remain underrepresented in preclinical studies. Our findings revealed that females were relatively less sensitive to the anxiogenic effects of CIE than males, showing no significant changes on the EPM or OFT. In contrast, females did show significant increases in anxiety-like behaviors on the SAT, perhaps reflecting the increased sensitivity of this assay. Importantly, we also identified a CIE-dependent increase in depressive-like behavior in females, as evidenced by an increase in immobility of CIE-treated females on the FST. As in males, CIE-dependent changes in hippocampal excitability were also generally restricted to the ventral subregion. Surprisingly, despite the fact that CIE did promote a negative affective state in female rats, vHC synaptic activity was actually decreased relative to controls. Moreover, unlike in males, an assessment of a variety of receptor and channel proteins known to play an integral role in regulating hippocampal excitability did not reveal any CIE-dependent expression level changes that might explain the observed decrease in vHC synaptic function. Collectively, these findings reveal important sex differences in both behavioral and neurobiological phenotypes that develop in a rodent model of alcohol dependence.

In individuals with AUD, acute withdrawal from heavy alcohol use is often associated with increases in anxiety and this negative affective state can precipitate relapse (Koob, 2014; Blaine et al., 2015; Koob and Volkow, 2016; Centanni et al., 2019). Similarly, animal models of this disorder, primarily conducted in male subjects, also promote robust increases in anxiety-like behaviors during withdrawal (Koob, 2014; Gilpin and Weiner, 2017). To assess the effects of CIE on anxiety phenotypes in female rats, we used a battery of assays that we recently employed in a similar study in male subjects. As in males, we found no effect of CIE on center time or locomotor activity in females in the OFT. However, while CIE significantly decreased open arm time and open arm entries in males on the EPM, these well-validated anxiety measures were unchanged after CIE in females. Prior studies in females, have yielded mixed results, with some (Devaud et al., 1999; Morales et al., 2015; Kasten et al., 2020) but not others (Tanchuck-Nipper et al., 2015; Henricks et al., 2017) identifying anxiogenic behavior on the EPM following withdrawal from CIE.

Other tests of anxiety-like behavior, such as novelty suppressed feeding, marble burying, and ultrasonic vocalizations have also revealed sex-dependent differences in CIE-treated rodents (Pleil et al., 2015; Henricks et al., 2017; Jury et al., 2017). These findings may well arise as anxiety is a complex, multidimensional construct and each assay may test unique components of the anxiety response (Ramos and Mormede, 1998). Therefore, observed sex differences in the effects of CIE on anxiety-like behaviors may reflect unique coping strategies between males and females rather than actual differences in how the sexes respond to CIE.

In an effort to further characterize the effects of CIE on anxiety measures in females, we also examined the effects of CIE on the SAT. This relatively underutilized assay creates a gradient of anxiety-like behavior by allowing rats to explore a four quadrant linear maze comprised of an enclosed, dark quadrant (least anxiogenic) and three open quadrants that get progressively brighter and narrower. This test may be more sensitive than the EPM and OFT in detecting changes in anxiety-like behavior (Deacon, 2013; Lahmann et al., 2014). In a prior study in male rats, we identified significant anxiogenic changes following CIE on this assay, including increased time in the enclosed arm and decreased head dips in the open zone adjacent to the enclosed arm (Ewin et al., 2019). Notably, in females, we observed a similar CIE-associated increase in time spent in the enclosed arm as well as a fourfold increase in latency to first egress from this zone. Interestingly, head dip frequency was not altered in any of the open arms (data not shown), underscoring the idea that males and females may indeed differ in the coping strategies that they employ on these assays.

What might be driving the discrepancies among the various anxiety assays, particularly in relation to sex-differences remains to be determined. However, the level of intoxication, the duration of CIE or withdrawal, the estrous cycle, the species and strain being tested, and potential differences in baseline anxiety-like behavior all likely play an important role.

In our present study, the average blood alcohol levels were somewhat lower than those achieved in other reports, including our previous study in male rodents (∼160 mg/dl vs. ∼210 mg/dl) (Lack et al., 2007; Morales et al., 2018; Ewin et al., 2019). Thus, we cannot rule out that these lower BECs impacted the behavior of our animals on some of the assays. It is important to note, however, that irrespective of the lower BECs, we did detect significant anxiety- and depressive-like behaviors in females. Thus, under our experimental conditions, CIE engendered a negative affective state in both male and female rats, despite the marked sex differences in vHC synaptic excitability observed during withdrawal. While most behavioral measures from control (air-exposed) rats were similar in females and males from our prior study, females did spend less time on the open arms than males, potentially hindering our ability to detect an anxiogenic effect of CIE with this measure. However, there were no sex differences in open arm entries and this measure of anxiety-like behavior was significantly altered by CIE in males but not females. In this study, we limited our investigation to a 10-day CIE paradigm with a 24-h withdrawal, a time point in which we and others have found this paradigm to show particularly robust behavioral and neurobiological changes (Lack et al., 2007; Morales et al., 2018; Ewin et al., 2019). Although most CIE studies have focused solely on male rodents, one study that compared the effects of different CIE exposure durations between male and female rats found that females required longer CIE exposure to develop some neurobiological adaptations in the BLA and measures of anxiety-like behavior (Morales et al., 2018). Future studies will be needed to determine if sex-dependent adaptations, as a function of the duration of CIE exposure and withdrawal, also influence CIE-associated neurobiological changes in the hippocampus. One important caveat to our present study is that all sex-dependent comparisons were based on our results from a prior study in males; male and female rats were not run side-by-side. It will be important, in future studies to conduct concurrent behavioral/neurobiological assessments in both sexes to control for possible environmental variables that may have impacted baseline (air-exposed control) or treated (CIE-exposed) measures.

We also expanded our exploration of CIE-dependent behavioral measures of negative affect by investigating depressive-like behavior using the FST. As with anxiety disorders, AUD is common in individuals suffering from comorbid depression and the moderating influence of depressive symptoms on risk of AUD may be greater in women. Most studies have reported that withdrawal from chronic alcohol exposure leads to increases in immobility on the forced swim test, a phenotype often interpreted to reflect increased depressive-like behavior. Interestingly, increased immobility has been reported in both male and female rodents, even with exposure durations as short as 3 h/day (Getachew et al., 2008, 2010, 2017). Consistent with these prior findings, we found a robust increase in immobility time in CIE-treated female rats. It will be important in future studies to conduct a more thorough analysis of CIE effects on depressive-like behaviors in males and females to determine if females may be more sensitive to the depressive phenotypes that develop during withdrawal from chronic alcohol exposure.

A striking finding from our CIE-treated female rats is that, while CIE primarily impacted synaptic excitability in the ventral domain of the hippocampus, as we had previously observed in males, CIE led to decreased, rather than increased synaptic excitability in this brain region. We found a decrease in both fV and fEPSP slope in CIE-treated female rats. While changes in fV are generally considered to reflect changes in presynaptic release, changes in fEPSP slope are indicative of postsynaptic adaptations. We did not, however, detect a difference the fEPSP slope normalized to the fV (fEPSP/fV) in female rats. This finding indicates that the coupling efficiency between presynaptic release and the magnitude of the resulting synaptic response generated postsynaptically is not changed between Air and CIE- treated rats. This could be due to adaptive processes that normalize postsynaptic responses decreasing presynaptic release (Simeone et al., 2013; Annamneedi et al., 2018; Reyes-Garcia et al., 2018). Overall, the evidence for functional plasticity leading to decreased neuronal excitation is, surprising given that we observed a negative affective state after CIE in females, a behavioral phenotype generally associated with enhanced vHC excitability. Indeed, many studies have shown that the vHC plays an integral role in anxiety-like behaviors and increases, rather, than decreases, in excitability in this hippocampal subregion are generally associated with anxiogenesis. A caveat is that the majority of these preclinical studies have been conducted in male subjects. In studies that have looked at sex-dependent neuronal response differences in models of alcohol dependence and/or stress, examples of sex-divergent responses are numerous (Morales et al., 2018; Blume et al., 2019; Dao et al., 2020; Joffe et al., 2020; Kasten et al., 2020). The BLA is a brain region of particular interest in this context, given that it sends a strong excitatory projection to the vHC that, when activated, elicits anxiogenesis (Felix-Ortiz and Tye, 2014; Beyeler et al., 2018). In the BLA, CIE results in an increase in neuronal excitability in both male and female rodents, but females require a more protracted exposure to CIE to develop this hyperexcitability (Morales et al., 2018). Basal activity of BLA neurons is significantly higher in female rats (Blume et al., 2017), and stress elicits opposite effects on neuronal firing patterns in males and females. While neurons of the BLA are hyperexcited in male rats, their activity is decreased in female rats in response to repeated stress (Blume et al., 2019). Similarly, in the vHC, stress has reliably been shown to increase neuronal activity in male rats (Chang et al., 2015; Chang and Gean, 2019; Wang et al., 2019). While present, this response is significantly blunted in female rats (Wang et al., 2019). Moreover, excitotoxic lesions of the vHC elicit anxiolytic responses in male rats (Kjelstrup et al., 2002; Bannerman et al., 2004), but do not alter behavior in female rats (Wang et al., 2019). Collectively, these findings suggest that there may be profound sex differences in both the circuitry that drives negative affective behaviors and the responsivity of this circuitry to repeated exposure to alcohol and stress. These emerging preclinical findings are mirrored by human studies of negative affective disorders revealing profound disease-related differences in gene expression between men and women (Labonte et al., 2017; Kang et al., 2020). Unfortunately, we were unable to identify any target proteins to explain the decrease in excitatory neurotransmission in the vHC of CIE treated female rats, despite exploring the role of a spectrum of targets commonly associated with changes in excitatory and inhibitory neurotransmission. There are several possible explanations for the absence of altered expression in our targeted proteins. One possibility is that the functional changes we observed may have reflected specific adaptations in distinct extrinsic (e.g., BLA) or intrinsic (CA3) inputs onto CA1 neurons and these inputs would have been intermixed using the methods employed to measure synaptosomal protein expression levels. Thus, protein expression levels within CIE insensitive synapses may have either masked or even counterbalanced changes in the expression of proteins in CIE-sensitive pathways. This explanation could also account for the absence of functional changes in dHC synapses despite modest changes in the expression of proteins that regulate both excitatory and inhibitory neurotransmission in dHC synaptosomes. Regions, including area CA2 of the dHC, make projections to the vHC (Tamamaki et al., 1988; Kohara et al., 2014). These projections open up the possibility that that functional changes in the vHC are, in part, dependent on modulation of the dHC. Another possibility that could explain the absence of expression level changes in vHC synaptic proteins is that CIE-dependent functional changes may be driven by posttranslational modifications, rather than alterations in protein levels. In the BLA, posttranslational changes (phosphorylation status) of AMPAR subunits are responsible for an increase in excitatory neurotransmission following CIE-treatment (Christian et al., 2012). Finally, although the synaptic proteins that we examined represent some of the most common targets involved in driving CIE-dependent changes in excitatory neurotransmission, it remains possible that other targets are responsible for the functional changes in vHC synaptic excitability that we observed in CIE-treated females. One target that is of particular interest for future studies is the GluN2A subunit. This subunit is highly expressed in the CA1 region of the hippocampus (Paoletti, 2011). Polymorphisms in this subunit have also been implicated in human subjects with alcohol dependence lending clinical relevance to the multitude of studies in rodent models that have pointed to the importance of the GluN2A subunit in alcohol dependence (Boyce-Rustay and Holmes, 2006; Schumann et al., 2008; Domart et al., 2012; Daut et al., 2015; Jury et al., 2018; Zamudio et al., 2020). In conclusion, our findings, along with those of many other preclinical studies exploring the neurobiology of AUD and negative affective states, highlight important sex differences in both the behavioral consequences of chronic alcohol exposure and the neural circuitry that likely drives these maladaptive behaviors. While many questions remain to be addressed, these studies strongly suggest that biological sex is critical factor to consider.

## Data Availability Statement

The original contributions presented in the study are included in the article/supplementary material, further inquiries can be directed to the corresponding author.

## Ethics Statement

The animal study was reviewed and approved by the Wake Forest University Animal Care and Use Committee.

## Author Contributions

JM and SB conducted the experiments. EB, JM, SB, and SE analyzed the data. EB, JW, and KR-G helped to design the experiments and guide and data analysis. EB and JW wrote and edited the manuscript. All authors contributed to the article and approved the submitted version.

## Conflict of Interest

The authors declare that the research was conducted in the absence of any commercial or financial relationships that could be construed as a potential conflict of interest.

## Abbreviations

AUD: alcohol use disorder
CIE: chronic intermittent ethanol
dHC: dorsal hippocampus
vHC: ventral hippocampus
EPM: elevated plus-maze
OFT: open field test
FST: forced swim test
BEC: blood ethanol concentrations
fEPSP: field excitatory post synaptic potential
fV: fiber volley
SN: synaptoneurosomal
SK2: small conductance calcium-activated potassium channel.
